# Arginine-Vasopressin Receptor Blocker Conivaptan Reduces Brain Edema and Blood-Brain Barrier Disruption after Experimental Stroke in Mice

**DOI:** 10.1371/journal.pone.0136121

**Published:** 2015-08-14

**Authors:** Emil Zeynalov, Susan M. Jones, Jeong-Woo Seo, Lawrence D. Snell, J. Paul Elliott

**Affiliations:** 1 Swedish Medical Center, Neurotrauma Research, Englewood, Colorado, United States of America; 2 Colorado Neurological Institute, Englewood, Colorado, United States of America; 3 Colorado Brain and Spine Institute, Englewood, Colorado, United States of America; Indian Institute of Integrative Medicine, INDIA

## Abstract

**Background:**

Stroke is a major cause of morbidity and mortality. Stroke is complicated by brain edema and blood-brain barrier (BBB) disruption, and is often accompanied by increased release of arginine-vasopressin (AVP). AVP acts through V1a and V2 receptors to trigger hyponatremia, vasospasm, and platelet aggregation which can exacerbate brain edema. The AVP receptor blockers conivaptan (V1a and V2) and tolvaptan (V2) are used to correct hyponatremia, but their effect on post-ischemic brain edema and BBB disruption remains to be elucidated. Therefore, we conducted this study to investigate if these drugs can prevent brain edema and BBB disruption in mice after stroke.

**Methods:**

Experimental mice underwent the filament model of middle cerebral artery occlusion (MCAO) with reperfusion. Mice were treated with conivaptan, tolvaptan, or vehicle. Treatments were initiated immediately at reperfusion and administered IV (conivaptan) or orally (tolvaptan) for 48 hours. Physiological variables, neurological deficit scores (NDS), plasma and urine sodium and osmolality were recorded. Brain water content (BWC) and Evans Blue (EB) extravasation index were evaluated at the end point.

**Results:**

Both conivaptan and tolvaptan produced aquaresis as indicated by changes in plasma and urine sodium levels. However plasma and urine osmolality was changed only by conivaptan. Unlike tolvaptan, conivaptan improved NDS and reduced BWC in the ipsilateral hemisphere: from 81.66 ± 0.43% (vehicle) to 78.28 ± 0.48% (conivaptan, 0.2 mg, p < 0.05 vs vehicle). Conivaptan also attenuated the EB extravasation from 1.22 ± 0.08 (vehicle) to 1.01 ± 0.02 (conivaptan, 0.2 mg, p < 0.05).

**Conclusion:**

Continuous IV infusion with conivaptan for 48 hours after experimental stroke reduces brain edema, and BBB disruption. Conivaptan but not tolvaptan may potentially be used in patients to prevent brain edema after stroke.

## Introduction

Stroke is the fourth leading cause of death and the first leading cause of disability in the US [1]. The onset of stroke is followed by life-threatening pathophysiological responses such as brain edema, blood-brain barrier (BBB) disruption, elevation of intracranial pressure with consequent reduction of cerebral blood flow (CBF) [2] and further progression of ischemic brain damage and secondary brain injury. Stroke is often complicated by uncontrolled secretion of arginine-vasopressin (AVP) and hyponatremia [3], which may have deleterious effects on the brain [4]. AVP activates V1a and V2 receptors [5] causing vasospasm [6], platelet aggregation [7], water retention, dilutional hyponatremia, and low plasma osmolality [8]. These events can quickly exacerbate stroke-evoked brain edema [9] and increase mortality rate [10] if plasma sodium level is not corrected. Therefore, blocking both V1a and V2 receptors after ischemic brain injury may prevent harmful pathophysiological events such as vasoconstriction, platelet aggregation, and hyponatremia.

Recent literature suggests that selective V1a receptor blockers can reduce post-ischemic and post-traumatic brain edema formation [11–13], provide neuroprotection [11], and decrease BBB disruption [14–16] in animals. Blocking of V1a receptors after subarachnoid hemorrhage leads to improvements in regional cerebral blood flow [17], reduction of mean arterial blood pressure [17], and alleviation of AVP-induced vasoconstriction [6]. The V2 receptor blockers have been shown to induce aquaresis, i.e. excretion of water through the kidneys which can correct dilutional hyponatremia [18] by increasing plasma sodium levels and osmolality [19]. Clinically available AVP receptor blockers conivaptan (intravenously administered V1a and V2 blocker) [20] and tolvaptan (orally administered V2 receptor blocker) [21] are used to correct dilutional hyponatremia. But the effect of these drugs on post-ischemic brain edema has not been studied. As a combined V1a and V2 receptor blocker, conivaptan may regulate both vascular tone and plasma sodium concentration simultaneously. This property of conivaptan may offer an advantage over tolvaptan which is known to regulate only plasma sodium levels. Therefore, we designed a clinically relevant study to 1) investigate whether conivaptan can minimize brain edema and protect BBB after experimental stroke, and 2) to compare effects of two different AVP blockers conivaptan and tolvaptan on post-ischemic brain edema. Here we used an experimental stroke model *in vivo* to evaluate changes in brain edema, BBB disruption, plasma and urine sodium and osmolality, and neurological deficits in mice after treatment with conivaptan or tolvaptan.

## Methods

Experiments were carried out in accordance with the guidelines of the National Institutes of Health for the care and use of animals in research and were approved by the Swedish Medical Center Animal Care and Use Committee.

### Experimental design

**Fig 1:**
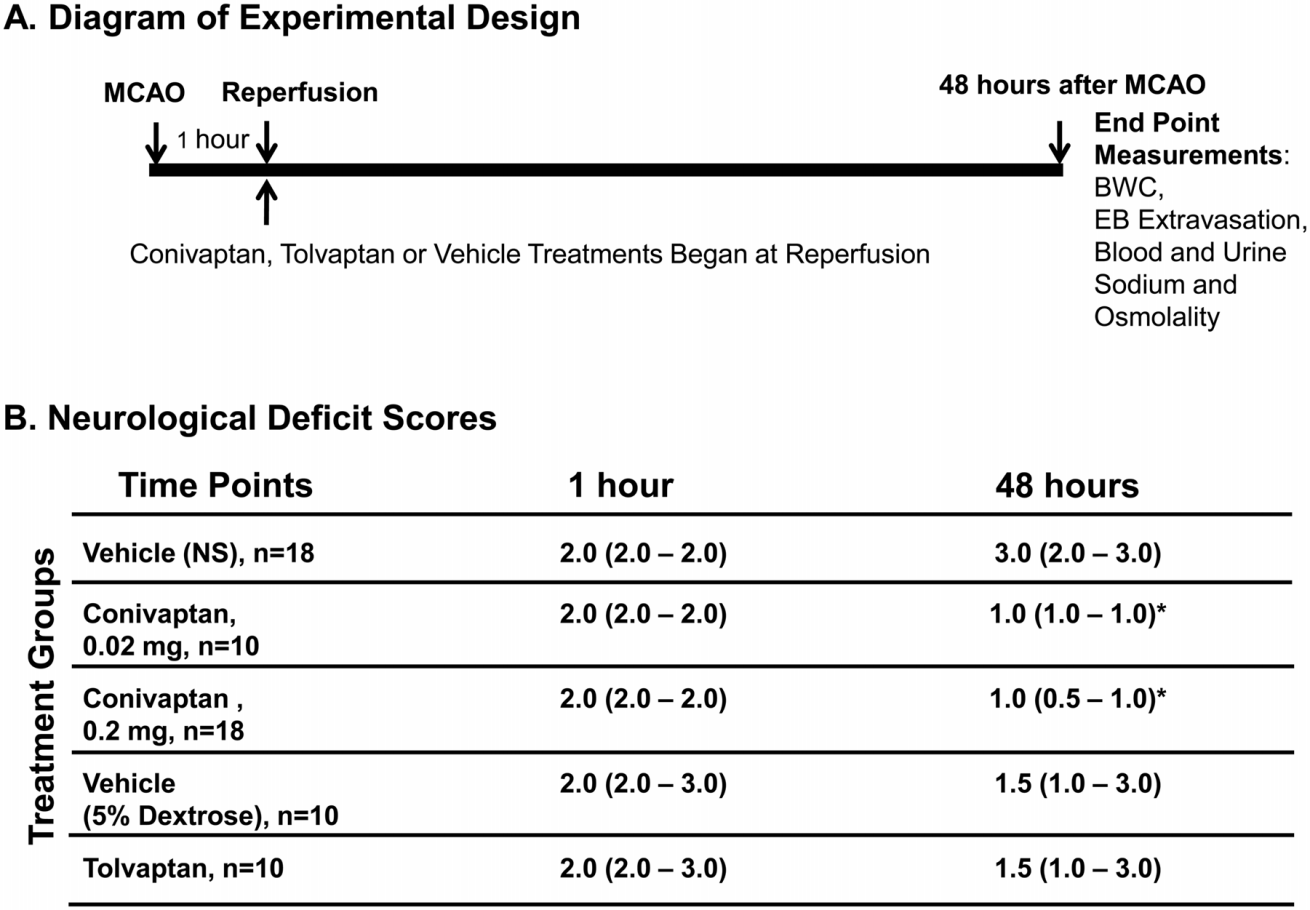
Diagram of experimental design and Neurological Deficits. (A) MCAO and reperfusion was performed surgically by the intraluminal filament technique. Conivaptan or tolvaptan treatment was initiated immediately at reperfusion (1 hour after MCAO) and lasted for 48 hours. At the end point (48 hours after MCAO) blood and urine was collected for sodium and osmolality measurements, and brains were used to quantify brain water content (BWC) or Evans Blue (EB) extravasation. (B) Neurological deficits were evaluated in all experimental animals twice: before reperfusion (1 hour after MCAO) and at the end point (48 hours after MCAO). Both doses of conivaptan improved NDS at 48 hours after MCAO as compared to vehicle-treated mice. Tolvaptan did not produce any improvements in NDS when compared to the vehicle-treated mice. NDS values are median (25%- 75%), *p < 0.05 vs. vehicle was considered significant.

We used wild type C57BL/6 male mice (Harlan Laboratories, Inc., Indianapolis, IN), 3 months old, 26–30 g (n = 82). Based on power analysis, we determined that 10 mice per group would be adequate to detect differences in brain water content (BWC). In two separate sets of experiments, mice were treated with either conivaptan or tolvaptan and compared to vehicle control. In a third set, eight mice per treated group and 5 naive mice were required to detect differences in blood brain barrier (BBB) disruption following conivaptan or vehicle treatment. Animals underwent transient focal brain ischemia by middle cerebral artery occlusion (MCAO) with reperfusion (Fig 1A). Treatments with conivaptan (Astellas Pharma US Inc, Deerfield, IL), tolvaptan (Tocris Bioscience, Bristol, UK), or appropriate vehicle were initiated immediately at reperfusion. During the time course of the experiment (48 hours) all mice had free access to food and water. At the end point of the experiment BWC or BBB disruption were assessed. To confirm the aquaretic effect we measured sodium and osmolality in plasma and urine in all experimental animals.

### MCAO and IV line installation

MCAO was performed as previously described [22]. Mice were anesthetized with 1–1.5% isoflurane in 25% O_2_-enriched room air delivered on spontaneous ventilation. Animals were placed in the supine position, and a midline incision was made along the neck. A silicone-coated 7.0 nylon filament was inserted through a small incision on the external carotid artery (ECA) and advanced through the internal carotid artery (ICA) about 7 mm towards the Circle of Willis to the point of origination of the middle cerebral artery (MCA). The wound was closed, and mice were awakened in the recovery cage placed on a heating pad to avoid hypothermia. Body temperature was maintained within the physiological range during MCAO and reperfusion times. Neurological deficit scoring (NDS) was evaluated by the same investigator and used to confirm successful occlusion as follows: 0 = normal motor function, 1 = flexion of torso and of contralateral forelimb on tail lift, 2 = circling to the contralateral side but normal posture at rest, 3 = leaning to contralateral side at rest, 4 = no spontaneous motor activity [22]. Mice that were scored less than 2 immediately after MCAO were excluded from the study. After 60 minutes the filament was removed to restore blood flow to brain. The IV line was installed immediately after the reperfusion. The IV line (Silastic Tubing, SIL-3-25, Strategic Applications, Inc., Lake Villa, IL) was inserted into the jugular vein 0.5 cm deep caudally and tied to the vein. Then the tubing was tunneled under the skin from the front to the back of the neck, then externalized and connected to infusion pump through swivels (375/22PS, Instech Laboratories, Inc. Plymouth Meeting, PA). Mice were moved to their home cage and recovered from anesthesia. NDS was measured again prior to conclusion of the study.

### Treatment

Mice were randomly allocated to the following treatment groups: conivaptan, 0.02 mg or 0.2 mg (IV), tolvaptan, 0.2 mg in 5% dextrose (oral), and vehicle for conivaptan (IV) or tolvaptan (oral). Continuous IV infusion with conivaptan or vehicle (normal saline) was initiated immediately at the onset of reperfusion. Conivaptan daily doses of 0.02 mg or 0.2 mg followed the loading dose (0.02 mg) and lasted 48 hours. In this study, we selected the 48-hour time frame based on recommendations for duration of continuous IV infusion of conivaptan in humans [23, 24]. Doses for mice were calculated based on conivaptan and tolvaptan human doses approved by the FDA [25, 26], adjusted for weight. The infusion rate for mice was 1.5 ml/kg/hour as previously described [22]. The selection of the IV infusion rate was based on our previous studies of continuous IV infusions in mice [22] as well as the considerations for safe daily fluid intake for mice [27]. Tolvaptan or vehicle (5% dextrose) were administered by the gavage method [28] twice a day. All experimental animals were used for blinded evaluation of BWC or BBB disruption at the end point (48 hours). We chose a 48 hour time point because our previous data suggested that MCAO-induced brain edema in mice peaks at 48–72 hours (unpublished). In addition, 5 naïve mice were used as controls for BBB evaluation.

### Evaluation of brain edema

Brain edema was assessed by comparing brain water content (BWC) between groups as described previously [22]. The brains were removed and separated into the ipsilateral and contralateral hemispheres. Hemispheres were weighed before and after they were dried in an oven for 3 days at 100°C. BWC was calculated as % H_2_O = (1-dry weight/wet weight) × 100.

### Evaluation of blood-brain barrier disruption

BBB permeability was assessed by the Evans blue (EB) extravasation method as previously described [22]. Evans Blue (2% in 0.9% saline; 3 ml/kg) was administered intravenously 2 hours prior to sacrifice. Via a thoracotomy under deep isoflurane anesthesia, intracardiac perfusion was then performed through the left ventricle with saline to remove intravascular EB dye, and continued until the fluid from the right atrium became colorless. Brains were quickly removed, and dissected into right (ischemic) and left (non-ischemic) hemispheres. Each hemisphere was weighed and homogenized in 2 ml of 50% trichloroacetic acid solution. After centrifugation at 3,000 g for 45 min, the supernatants were diluted with ethanol (1:3), and EB concentration was determined with a spectrophotometer at 620 nm for absorbance against a standard curve. BBB disruption data is presented as EB extravasation index which is the ratio of EB concentration in the ischemic hemisphere to that in the non-ischemic hemisphere (I/C).

### Assessment of plasma and urine sodium and osmolality

After the 48-hour treatment, mice were over-anesthetized with isoflurane; blood was aspirated directly from the heart and urine from the bladder. Sodium levels in plasma and urine were measured using Orion Star Series Meter with ROSS Sodium Ion Selective Electrode (Thermo Scientific, Waltham, USA). Osmolality (mOsmol/L) of plasma and urine samples were evaluated using a vapor pressure osmometer (VAPRO 5520; WESCOR, Inc, Logan UT).

### Statistical analysis

Values for BWC and EB extravasation index are expressed as mean ± SEM. BWC was analyzed by two-way ANOVA (treatment and hemisphere as factors) with post hoc Bonferroni test. EB extravasation index was analyzed by one-way ANOVA with Dunnett’s post-hoc test. Plasma and urine sodium and osmolality were analyzed by unpaired t-test. Neurological Deficit Score is presented as median (with 25% and 75% quartiles) and analyzed by the non-parametric Kruskal-Wallis with Dunns post-hoc test. For all analyses, differences were considered significant at *p* < 0.05.

## Results

### Conivaptan improves neurological deficits after stroke

A total of 11 mice were excluded from the study: two mice had NDS of 1 after MCAO and therefore were excluded. Nine mice died during the 48-hour treatment period after MCAO/reperfusion, but the mortality rate did not differ among groups (Table 1). Body temperature during MCAO and reperfusion was within the physiological range, and body weights were measured before surgery and at the end point of the experiment (48 hours). No significant differences were recorded between the compared groups, Tables 1 and 2. Neurological deficit scores (NDS) were improved in conivaptan but not in tolvaptan-treated treated mice, Fig 1B.

**Table 1:** Summary of physiological variables of animals treated with conivaptan.

	Vehicle	Conivaptan	Conivaptan
		0.02 mg	0.2 mg
**N**	18	10	18
**Body Temperature, °C**			
Occlusion	36.6 ± 0.1	36.5 ± 0.2	36.5 ± 0.1
Reperfusion	36.3 ± 0.3	36.8 ± 0.2	36.7 ±0.1
**Body Weight, g**			
0 hours	26.5± 0.9	26.1 ± 0.8	27.4 ± 0.7
48 hours	24.6 ± 1.0	23.3 ± 0.9	23.4 ± 0.7
**Mortality**	3/21	2/12	3/21

All values are mean ± SEM.

**Table 2:** Summary of physiological variables of animals treated with tolvaptan.

	Vehicle	Tolvaptan
**N**	10	10
**Body Temperature, °C**		
Occlusion	36.0 ± 0.12	36.1 ± 0.07
Reperfusion	35.9 ± 0.07	36.0 ± 0.12
**Body Weight, g**		
0 hours	27.7 ± 0.5	27.4 ± 0.5
48 hours	24.2 ± 0.6	24.2 ± 0.5
**Mortality**	0/10	1/11

All values are mean ± SEM.

### Conivaptan reduced brain water content in the ipsilateral and contralateral hemispheres

**Fig 2:**
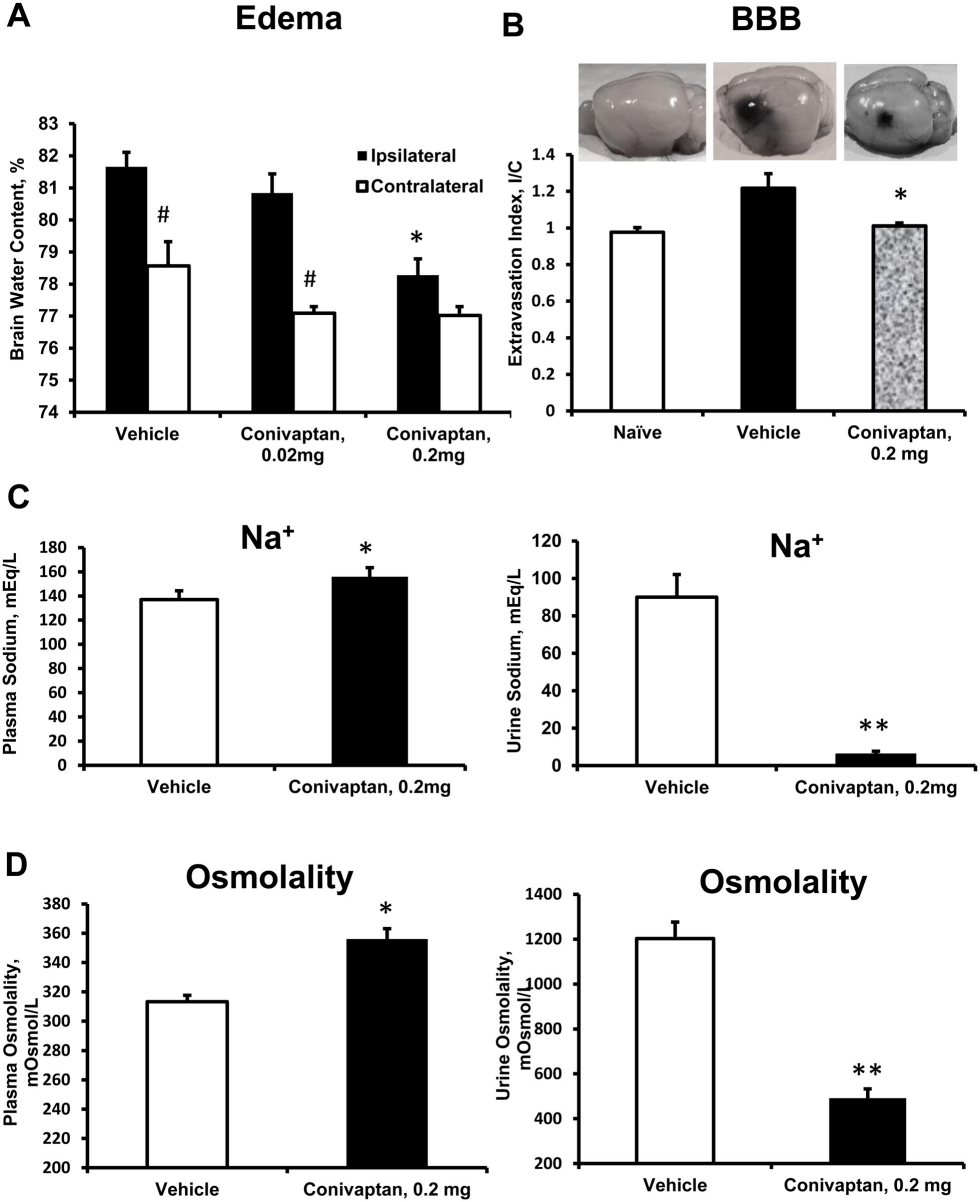
Effects of conivaptan on brain water content (BWC), blood-brain-barrier (BBB) disruption, plasma and urine sodium and osmolality. (A) Continuous IV infusion with conivaptan reduces stroke-evoked brain edema. A normalization of brain water content (BWC) in the ipsilateral hemisphere was observed in mice treated with conivaptan for 48 hours after MCAO/reperfusion at the dose 0.2 mg/day but not 0.02 mg/day. The slight increase in BWC of the contralateral hemisphere was also reduced by both doses of conivaptan, n = 10 in each group. (B) Continuous IV treatment with conivaptan for 48 hours decreased blood-brain barrier (BBB) disruption after 60-minute MCAO/reperfusion. Following MCAO, Evans Blue (EB) extravasation index (I/C) was increased in vehicle treated mice, and reduced in conivaptan treated mice, naïve, n = 5, vehicle, n = 8, Conivaptan 0.2 mg, n = 8. (C, D) Conivaptan treatment of 0.2 mg resulted in an elevation of plasma sodium and osmolality, and decreased urine sodium and osmolality due to aquaresis. All values are presented as mean ± SEM. *p < 0.05 and **p < 0.01 compared to vehicle, and ^#^p < 0.05 compared to ipsilateral hemisphere.

**Fig 3:**
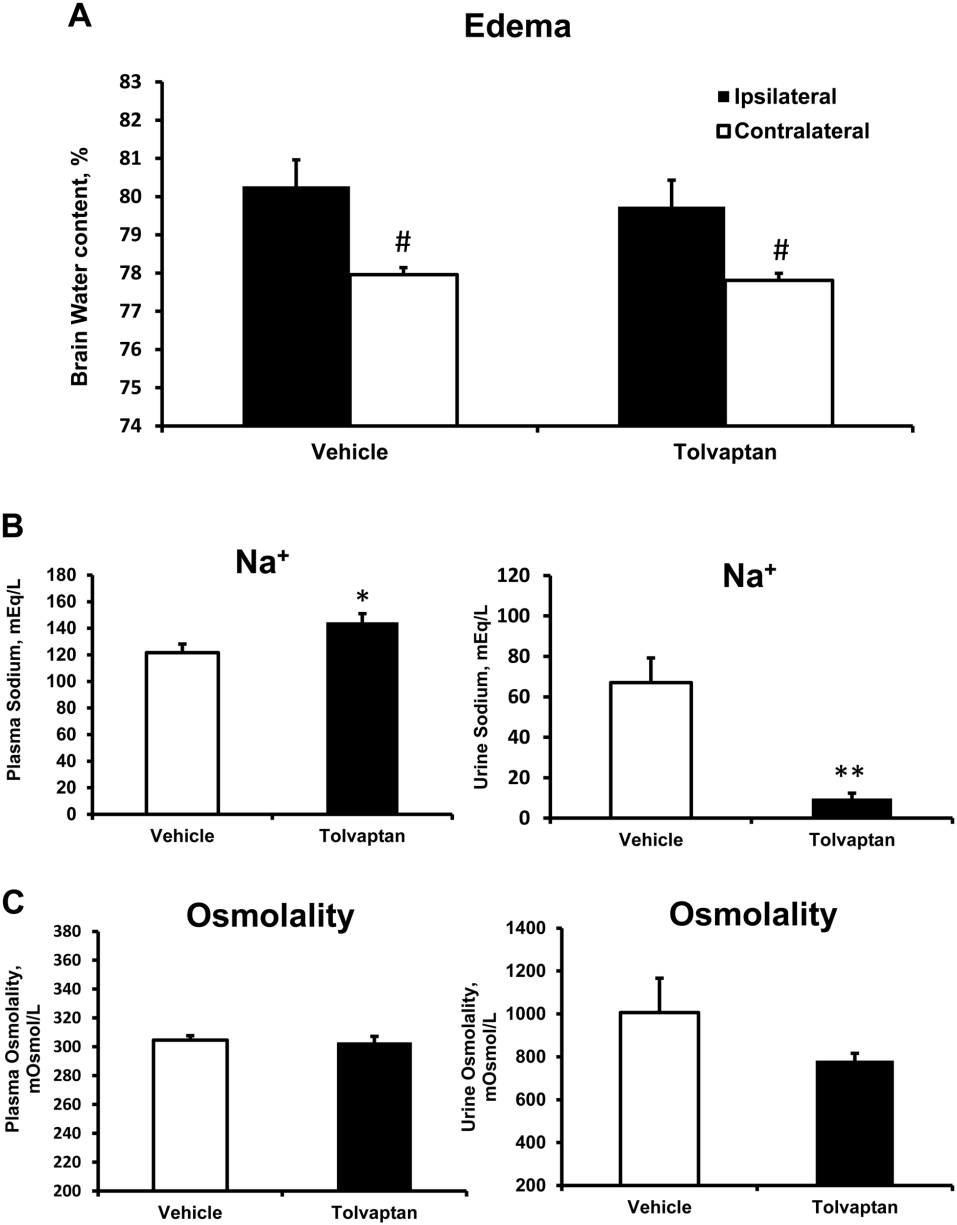
Effects of tolvaptan on brain water content (BWC), plasma and urine sodium and osmolality. (A) Tolvaptan treatment did not change BWC in mice. (B) Tolvaptan treatment causes aquaresis in mice as seen by increases in plasma sodium and decreases in urine sodium concentrations. (C) However, plasma and urine osmolality was unchanged by tolvaptan. All values are mean ± SEM. ^#^p < 0.05 compared to ipsilateral hemisphere, *p < 0.01 and **p < 0.0001 compared to vehicle, n = 10 in each group.

MCAO resulted in an increase in BWC which was greater on the ipsilateral side of the brain (Figs 2A and 3A). Continuous IV treatment with conivaptan 0.2 mg significantly reduced BWC (%) in both hemispheres: 78.28 ± 0.48 and 76.91 ± 0.27 in conivaptan 0.2 mg versus 81.66 ± 0.43 and 78.67 ± 0.71 in vehicle for ipsilateral and contralateral hemispheres respectively (Fig 2A). Conivaptan treatment at 0.02 mg significantly reduced BWC on the contralateral side only (77.08 ± 0.2). Treatment with tolvaptan did not produce changes in BWC of ipsilateral or contralateral hemispheres: 79.74 ± 0.44 and 77.82 ± 0.10 in tolvaptan versus 80.27 ± 0.69 and 77.96 ± 0.18 in vehicle for ipsilateral and contralateral hemispheres respectively (Fig 3A).

### Evans Blue extravasation index was reduced in conivaptan-treated mice

MCAO increased the Evans Blue extravasation index (I/C) in mice treated with vehicle compared to naïve untreated mice. Conivaptan treatment at 0.2 mg significantly reduced MCAO-evoked BBB disruption. I/C values were as follows: 0.98 ± 0.03 (naïve); 1.22 ± 0.07 (vehicle); and 1.01 ± 0.02 (conivaptan 0.2 mg), Fig 2.

### Conivaptan treatment changed plasma and urine sodium and osmolality

Both conivaptan and tolvaptan have demonstrated aquaretic properties in mice. Conivaptan treatment increased plasma sodium concentration (in mEq/L) from 137.0 ± 7.4 (vehicle) to 155.9 ± 7.5 (conivaptan 0.2 mg), and decreased urine sodium from 90.0 ± 12.1 (vehicle) to 6.1 ± 1.6 (conivaptan 0.2 mg), Fig 2C. Similarly, tolvaptan resulted in increased plasma sodium from 121.6 ± 6.4 (vehicle) to 144.5 ± 6.5 (tolvaptan), and decreased urine sodium from 67.0 ± 12.2 (vehicle) to 9.7 ± 2.7 (tolvaptan), Fig 3B. The changes in sodium concentrations of conivaptan-treated mice were mirrored by osmolality changes (mOsm/L) in plasma and urine respectively: 313.2 ± 4.4 and 1210.6 ± 130.4 4 (vehicle), 355.6 ± 7.5 and 592.0 ± 57.4 (conivaptan, 0.2 mg), Fig 2D. Tolvaptan treatment in mice did not affect plasma and urine osmolality respectively: 304.7 ± 3.0 and 1006.6 ± 160.7 (vehicle) compared to 302.7 ± 4.5 and 782.7 ± 33.8 (tolvaptan), Fig 3C.

## Discussion

This study reveals important findings that can be useful in treating patients with ischemic brain injury: 1) Conivaptan, a V1a and V2 receptor blocker, reduces stroke-evoked brain edema formation and BBB disruption, and improves neurological deficits in mice; 2) Tolvaptan, a V2 receptor blocker, on the other hand, has no effect on post-ischemic brain edema and neurological deficits. This newly described feature of conivaptan can be translated into clinical applications to improve outcomes after stroke.

Earlier studies showed that V1a receptor blockade is responsible for reversing vasoconstriction [29] and platelet activation [7], and was reported to improve regional CBF after subarachnoid hemorrhage [17]. V1a receptor blockers have been shown to attenuate brain water content in mice after stroke when administered via intracerebroventricular injection [11, 16]. V1a receptor blockers have also been shown to reduce post-ischemic and post-traumatic brain edema in rodents [11–13, 30, 31]. However, all of these previous studies used drugs that are unavailable for clinical application. To inspire a quick translation of these findings into treatment of brain edema in the ICU settings, we explored the effects of conivaptan, a clinically available V1a and V2 receptor blocker, on brain edema formation. Because conivaptan has equal affinity for V2 receptors, we also explored the effects of tolvaptan, another clinically available drug that blocks V2 receptors. A recent clinical case reported a reduction of severe stroke-induced brain edema by conivaptan, providing proof that the mixed AVP receptor blocker can be beneficial even if other therapeutic interventions are ineffective [32]. Based on the presented case, conivaptan administration was safe in the patient with normal base line plasma sodium concentration [32].

The dose selections for conivaptan and tolvaptan were based on the FDA recommendations for humans [23, 26]. However, the translated human-to-mouse conivaptan loading and daily dose (0.02 mg) effectively changed plasma and urine osmolality (not shown) but did not affect BWC, possibly due to the differences in the rate of drug metabolism between human and mice. After increasing the daily dose 10 times (0.2 mg) we were able to produce desirable results in reducing brain edema and BBB disruption, when a significant elevation in plasma sodium and osmolality was also achieved.

The dose for FDA approved oral route of administration of tolvaptan was chosen in a similar fashion (x10 of the human dose) and showed sufficient aquaretic effect with no signs of toxicity in experimental animals. Nevertheless, tolvaptan failed to reduce brain edema after stroke, which suggests that conivaptan may be a more suitable AVP receptor blocker to treat brain edema.

Both conivaptan and tolvaptan exhibit the V2 receptor—blocking effect (aquaresis) which removes excess water from the body [33] and cause elevation of plasma sodium (Figs 2C and 3B). In our study plasma sodium levels in vehicle-treated groups of mice after stroke were within the physiological range. Treatment with either conivaptan or tolvaptan increased plasma sodium concentrations to tolerable levels and indicates that the dose (0.2 mg) produces an aquaretic (V2 blocking) effect in mice. However, the increase in plasma sodium concentration was correlated with increases in plasma osmolality and reduction of brain edema only in conivaptan, but not tolvaptan treated animals. We speculate that this discrepancy in effect on plasma osmolality between the two drugs is due to the differences in routes of administration: continuous IV for conivaptan versus oral for tolvaptan. Elevation of plasma osmolality may be crucial for the osmotic effect to reduce brain edema because it creates an osmotic gradient between vascular and cellular compartments [34], which drives water from the brain cells by osmotic forces [34]. Continuous IV infusion of conivaptan, unlike oral delivery of tolvaptan, produces a steady plasma drug concentration to elevate plasma sodium and osmolality and provide greater benefit in reduction of edema. Therefore, we speculate that inability of tolvaptan to elevate plasma osmolality is due to the oral treatment which is the only route suggested by the FDA and the manufacturer. Tolvaptan’s low affinity for V1a receptors, as well as the inability to maintain an osmotic gradient in the plasma could explain the ineffectiveness of tolvaptan at reducing post-ischemic brain edema.

The aquaretic effect of conivaptan caused a slight decrease of brain water content on the contralateral (non-ischemic) side which is shown to be without any consequences for mice. Our previously published data of hyperosmolar therapy in mice after stroke confirms that a slight reduction within the physiological range of BWC in the contralateral hemisphere is well tolerated [22]. Moreover, this reduction of BWC on the contralateral side may be helpful in overall reduction of intracranial pressure (ICP) after stroke which might be beneficial for the ischemic hemisphere as well. The ability of a V1a receptor blocker to reduce ICP in animals has been demonstrated [17], and clinical studies of conivaptan have shown the same effect on ICP [35, 36].

In addition to a reduction of edema, we have demonstrated that conivaptan can protect BBB after stroke. Ischemic stroke causes breakdown of BBB, and AVP’s actions on V1a receptors may play an important role in BBB integrity [16, 37, 38]. It is also possible that the protective effect of conivaptan on BBB may be amplified due to combined V1a and V2 blocking effects of conivaptan: simultaneous regulation of cerebrovascular tone and plasma sodium levels and osmolality. The subsequent increase in plasma sodium and osmolality may attenuate brain edema, improving cerebral blood flow to the post-ischemic brain region and preventing the secondary brain injury and BBB disruption. However, in our study the V2 receptor blocker tolvaptan failed to reduce stroke-evoked brain edema, NDS and plasma osmolality. Therefore, we did not pursue further experiments to evaluate whether tolvaptan can improve BBB integrity after stroke.

In designing our study we were guided by importance of clinical relevance. Conivaptan is efficient in humans when delivered by continuous IV infusion [39]. In our study continuous IV infusion of conivaptan has proven to be most effective in reducing brain edema, and other routes of administration in mice (IP, SQ) did not affect stroke evoked brain edema [40]. Many animal studies with selective V1a or V2 receptor antagonists successfully use intra-cerebroventricular [11] as well as IP routes [41]. However, in our study we treated mice with FDA approved drugs using clinical routes of administration. In choosing the time for initiation of the treatment in our model (1 hour of ischemia) we first needed to demonstrate that conivaptan can alleviate stroke-evoked brain edema at early time points after stroke. Then, it would be more appropriate to investigate the effects of delayed conivaptan treatment. We are currently working to explore therapeutic time window for conivaptan administration because most ICU patients are delivered to the hospital hours after the onset of stroke. Our study also indicates that continuous IV infusion of Conivaptan can be safely applied in mice following MCAO. Conivaptan did not cause mortality at either 0.02 mg or 0.2 mg doses, and was observed to be well tolerated by our mice. All animals had full access to drinking water and food during the treatment, and we did not observe treatment-related mortality or behavior or any signs of dehydration or malnutrition that would indicate toxicity.

It remains to be determined whether conivaptan or tolvaptan are able to access the ischemic core after stroke, and to what degree they penetrate the penumbra region. It also remains to be demonstrated whether it is necessary for conivaptan to get to the extracellular space to produce the desired changes in recovery after stroke. The ischemic site of the brain that has decreased blood flow due to obstruction or vasoconstriction may not receive sufficient amount of conivaptan to produce local effects, such as prevention of vasoconstriction and removal of excess water from the tissue. This may explain why low dose conivaptan (0.02 mg) reduced BWC on the contralateral side, but a significant effect on the ipsilateral side required a higher dose.

This translational study can potentially improve care of stroke patients as it opens new possibilities for use of conivaptan. Future studies will be necessary to determine the optimal regimen for conivaptan treatment, as well as whether the treatment can provide with long-term benefits for ICU patients with brain edema.

## Conclusions

This study supports our hypothesis that blockade of both AVP receptors can be beneficial in preventing stroke-evoked brain edema and BBB breakdown. Continuous IV infusion of the V1a and V2 receptor blocker conivaptan reduces brain edema and BBB disruption, and improves neurological deficits in mice 48 hours after experimental focal ischemia. Tolvaptan, a V2 receptor blocker, has no effect on post-ischemic brain edema formation in mice. These effects of conivaptan on post-ischemic brain edema and BBB permeability have great translational value because conivaptan is clinically available. Most importantly, conivaptan should be considered for studies of other neurological diseases that lead to brain edema and BBB disruption.
